# A Two-Factor Model Better Explains Heterogeneity in Negative Symptoms: Evidence from the Positive and Negative Syndrome Scale

**DOI:** 10.3389/fpsyg.2016.00707

**Published:** 2016-05-12

**Authors:** Seon-Kyeong Jang, Hye-Im Choi, Soohyun Park, Eunju Jaekal, Ga-Young Lee, Young Il Cho, Kee-Hong Choi

**Affiliations:** ^1^Department of Psychology, Korea UniversitySeoul, South Korea; ^2^Department of Psychology, Sungshin Women’s UniversitySeoul, South Korea

**Keywords:** schizophrenia, psychosis, negative symptoms, subdomain, expressive deficits, experiential deficit

## Abstract

Acknowledging separable factors underlying negative symptoms may lead to better understanding and treatment of negative symptoms in individuals with schizophrenia. The current study aimed to test whether the negative symptoms factor (NSF) of the Positive and Negative Syndrome Scale (PANSS) would be better represented by expressive and experiential deficit factors, rather than by a single factor model, using confirmatory factor analysis (CFA). Two hundred and twenty individuals with schizophrenia spectrum disorders completed the PANSS; subsamples additionally completed the Brief Negative Symptom Scale (BNSS) and the Motivation and Pleasure Scale—Self-Report (MAP-SR). CFA results indicated that the two-factor model fit the data better than the one-factor model; however, latent variables were closely correlated. The two-factor model’s fit was significantly improved by accounting for correlated residuals between N2 (emotional withdrawal) and N6 (lack of spontaneity and flow of conversation), and between N4 (passive social withdrawal) and G16 (active social avoidance), possibly reflecting common method variance. The two NSF factors exhibited differential patterns of correlation with subdomains of the BNSS and MAP-SR. These results suggest that the PANSS NSF would be better represented by a two-factor model than by a single-factor one, and support the two-factor model’s adequate criterion-related validity. Common method variance among several items may be a potential source of measurement error under a two-factor model of the PANSS NSF.

## Introduction

Negative symptoms are an important dimension of schizophrenia symptoms, and are independent of positive and disorganized symptoms; negative symptoms have under-known pathological mechanisms and few treatment options (Harvey et al., 2006; Marder et al., 2011). To advance knowledge of negative symptoms’ potential pathology and means of intervention, valid and reliable measurement of negative symptoms is mandatory. Measurement requires assessment tools that accurately reflect the symptoms’ conceptualization. Recently, a consensus has emerged on the existence of multiple dimensions of negative symptoms (Blanchard and Cohen, 2006). Specifically, avolition (lack of interest in daily activities), asociality (reduced social interest and social withdrawal), and anhedonia (reduced ability to experience or anticipate pleasure) comprise the experiential deficits of negative symptoms, which may be correlated with, but are distinct from, expressive deficits such as blunted affect (diminished facial expression) and alogia (poverty of speech; Kirkpatrick et al., 2006). This two-factor structure has been verified in the course of the development of new assessments of negative symptoms and is reflected in the renewed conceptualization of negative symptoms in the Diagnostic and Statistical Manual of Mental Disorders, Fifth Edition (Messinger et al., 2011; Strauss et al., 2012; Kring et al., 2013).

The proposition that negative symptoms are composed of more than a single dimension has important implications: different dimensions might possess different underlying causes, courses, or treatment responses. Consistent with this idea, recent research suggests that the two dimensions mentioned above may have distinct clinical and functional correlates (Galderisi et al., 2013; Lyne et al., 2014; Quinlan et al., 2014). For example, Galderisi et al.’s (2013) 5-year longitudinal study found that “avolition” and “poor emotional expression” each predicted different aspects of social functioning outcomes: the former predicted social contact; the latter predicted household activities. Similarly, Ergül and Üçok (2015) found that “expressive” and “motivation/pleasure” deficits had distinct clinical correlates; for instance, the former was associated with earlier onset, lower education, and poorer performance in cognitive tests, and the latter with duration of untreated psychosis and family history of psychosis. Additionally, Cella et al. (2014) reported that detailed symptom dimensions can be used to increase the sensitivity of treatment response evaluations. This suggests that separate assessment of two dimensions would provide such benefits as more sensitive treatment-outcome measures and increased opportunity to investigate the distinct pathophysiology underlying the potentially separable negative symptom dimensions.

The Positive and Negative Syndrome Scale (PANSS) is widely used to measure psychiatric symptoms and provides valid and useful information about negative symptoms (Kirkpatrick et al., 2006). Recent research has reported that the PANSS negative symptoms factor (NSF) has greater content validity than the original negative symptoms scale, and is reliable, valid, and sensitive to treatment responses (Kirkpatrick et al., 2006; Edgar et al., 2014). Few studies have examined if the NSF has a similar two-factor structure to second-generation instruments for negative symptoms such as the Clinical Assessment Interview for Negative Symptoms (CAINS) and the Brief Negative Symptom Scale (BNSS). Among earlier measures, the two-factor structure was identified in the Scale for the Assessment of Negative Symptoms (Kelley et al., 1999) and Schedule for Deficit Syndrome (Galderisi et al., 2013). Notably, one recent study proposed a two-factor structure within the NSF using exploratory factor analysis (EFA; Fervaha et al., 2014). It is therefore timely to determine if the PANSS NSF is best represented by a two- or single-factor structure, and examine its external validity with novel negative symptoms scales.

We aimed to examine the factor structure of the NSF in Korean individuals with chronic schizophrenia by comparing the fitness of the two-factor and single-factor models. We also aimed to establish the two-factor model’s external validity by investigating its agreement with the BNSS and MAP-SR. Demographic, clinical, and neurocognitive correlates of the two factors were examined.

## Materials and Methods

### Participants

Our sample included 220 patients (194 outpatients, 26 inpatients; 128 males, 92 females) diagnosed with schizophrenia, schizoaffective disorder, or psychotic disorder not otherwise specified according to the Diagnostic and Statistical Manual of Mental Disorders-IV. The primary diagnosis was confirmed using the Korean version of the Mini-International Neuropsychiatric Interview-Plus for outpatients and the Structured Clinical Interview for DSM-IV Axis I Disorders for inpatients (Lecrubier et al., 1997; First et al., 2012). Participants were recruited from community mental health centers and an inpatient psychiatric hospital. All participants but one were stably on antipsychotic medications. Participants were excluded if they met criteria for brain injuries, developmental disorders, histories of substance abuse, or neurological disorders. Written informed consent was obtained before participation. This study was approved by the local institute review board of Korea University.

### Procedure

Data were collected in the context of several research projects, including a validation trial of the BNSS, efficacy trials of psychosocial rehabilitation programs, and experimental studies on negative symptoms. Participants were administered the PANSS at the start of the study by five raters (four master’s-level and one doctoral-level in clinical psychology) who had been trained using the PANSS’ educational materials and whose inter-rater reliability had been established (α = 0.76). A subsample of participants was additionally administered the MAP-SR (*n* = 141) and BNSS (*n* = 78) by three raters (two master’s-level and one doctoral-level in clinical psychology). These raters were trained using the original BNSS manual; inter-rater reliability was α = 0.83.

### Measures

#### Positive and Negative Symptom Scale (PANSS)

The PANSS measures comprehensive psychiatric symptoms, including positive, negative, and general symptoms (Kay et al., 1987). The PANSS includes 30 items; responses used a 7-point Likert scale and were given in semi-structured interviews. Yi et al. (2001) reported that the internal consistency of the Korean version of the PANSS is α = 0.73, α = 0.84, and α = 0.74 for the positive, negative, and general psychopathology scales, respectively. In the current study, the Cronbach’s alpha of the NSF (seven items: N1, N2, N3, N4, N6, G7, and G16) was 0.90; the inter-rater reliability of our trained raters was 0.76.

#### Motivation and Pleasure Scale—Self Report (MAP-SR)

The MAP-SR was used to measure the experiential deficits of negative symptoms such as anhedonia and amotivation (Llerena et al., 2013). This is a self-report measure including 15 items and developed based on the CAINS. Lower scores indicate low pleasure and motivation in social, work, and recreational domains. It measures diverse aspects of hedonic and motivational experiences including retrospective and anticipatory pleasure, and motivation and efforts to engage in such activities. The Cronbach’s alpha of the MAP-SR in this study was 0.92.

#### Brief Negative Symptom Scale (BNSS)

The BNSS is a newly developed semi-structured clinical interview assessment that measures the severity of negative symptoms in schizophrenia and schizoaffective disorder (Strauss et al., 2012). The BNSS is comprised of six subscales: anhedonia, asociality, avolition, blunted affect, alogia, and lack of general distress. There are 13 items in total, which are rated on a 7-point scale ranging from the absence of symptoms (0) to extremely severe symptoms (6). Symptoms are rated in terms of severity in the past week. Previous studies have found a two-factor structure: expressive deficits, comprised of the blunted affect and alogia subscales, and experiential deficits, comprised of the anhedonia, asociality, and avolition subscales. It has exhibited good internal consistency (α = 0.94) and discriminant and convergent validity (Strauss et al., 2012). In this study, the Korean version of the BNSS showed good internal consistency (α = 0.94) and the two-factor structure (expressive and experiential deficits factors, correlation: α = 0.80) of the original study exhibited adequate data fit: χ^2^ = 77.41, df = 49 (*p* < 0.01), NC = 1.58, CFI = 0.96, TLI = 0.95, RMSEA = 0.086 [CI: 0.047–0.121, 90%], and SRMR = 0.056.

#### Trail Making Test Parts A and B (TMT-A/B)

The TMT-A/B was included to examine basic cognitive function’s relationship with two dimensions of negative symptoms. In part A of the Trail Making Test (TMT-A), subjects connected scattered numerals ranging from 1 to 25 in numerical order. This part measured psychomotor speed and attention. In part B (TMT-B), subjects connected 15 numerals and 14 letters alternately in ascending order. This part measured executive functions (e.g., mental flexibility), which are related to frontal lobe functioning (Reitan, 1958). Time taken to complete each task was used in the analysis.

### Analysis

#### Confirmatory Factor Analysis (CFA)

Confirmatory factor analysis was conducted using a two-factor model identified in EFA in the past study (Fervaha et al., 2014). We chose to use CFA instead of EFA because we already had a theoretical framework, an existing model proposed through empirical research, and an alternative model to compare. Specifically, seven NSF items (N1, blunted affect; N2, emotional withdrawal; N3, poor rapport; N4, passive social withdrawal; N6, lack of spontaneity and flow of conversation; G7, motor retardation; G16, active social avoidance) were employed as observed variables. The fitness of the model in which the expressive deficit factor was represented by N1, N2, N3, N6, and G7 and experiential deficit factor represented by N2, N4, and G16 was subsequently computed and evaluated. The fit of the model was also compared with that of one-factor model, in which the seven NSF items were loaded onto one underlying factor. The maximum likelihood method was used for estimation as the data did not show any great tendency to non-normality (skewness < 2.00, Kurtosis < 7.00; West et al., 1995). Values of multiple indices of goodness-of-fit were computed and used for model evaluation: chi-square, comparative fit index (CFI values of >0.90 are indicative of acceptable fit), Tucker–Lewis index (TLI > 0.90), root mean square error of approximation (RMSEA < 0.10), and standardized root mean square residual (SRMR < 0.08; MacCallum et al., 1996; Hu and Bentler, 1999). As the value of chi-square is greatly affected by sample size, the normed chi-square (NC) was also calculated by dividing the chi-square value by the corresponding degrees of freedom (Bagozzi and Yi, 1988). An NC of less than 5.00 is considered good (Schumacker and Lomax, 2004). The models were also compared using chi-square difference tests (Bollen, 2014). CFA and chi-square difference tests were conducted using Mplus 6.1 and the lavaan package implemented in R x64 3.1.0 (Rosseel, 2012).

#### Correlational Analysis

Pearson correlation coefficients were calculated between the two factors (composite score) of the NSF and demographic variables. Demographic variables included age, gender, years of education, age of onset, and illness duration. Regarding gender, point-biserial correlations were calculated using dummy variables (0 = male, 1 = female). The external validity of the two NSF factors was assessed by analyzing these two factors’ specific relationship with expressive and experiential deficits in other measures of negative symptoms, and with cognitive function. Specifically, the two NSF factors’ correlation with the MAP-SR, the two subdomains of the BNSS, and the TMT-A/B was calculated using Pearson’s *r.* Williams’ test (implemented in R x64 3.1.0) was used to test the significance of differences in correlation coefficients’ magnitude (Steiger, 1980).

## Results

### Demographic and Clinical Information

Demographic data indicated that our sample was mostly composed of patients with a primary diagnosis of schizophrenia. Most participants were chronic patients with a mean illness duration of 15.85 ± 9.57 years and generally mild symptoms. All participants except one were taking antipsychotics. Full demographic characteristics are presented in **Table 1**. The MAP-SR and BNSS were additionally administered to subsamples (*n* = 141; *n* = 78, respectively). No significant differences existed regarding age, gender, education, or PANSS symptoms between subsamples and the full sample (*p* > 0.05).

**Table 1:** Demographic and clinical information.

		Participants (*n* = 220)
Primary diagnosis	Schizophrenia	201
	Schizoaffective	9
	Psychotic disorder NOS	10
Age	Mean (*SD*)	41.20 (10.47)
Gender (male)	%	58.20
Years of education^1^	Mean (*SD*)	12.45 (2.54)
Age of onset^2^	Mean (*SD*)	24.01 (8.14)
Duration of illness^2^	Mean (*SD*)	16.56 (9.80)
Antipsychotics	Medicated	219
	Non-medicated	1
PANSS positive^3^	Mean (*SD*)	2.59 (1.01)
PANSS negative	Mean (*SD*)	2.63 (1.01)
PANSS disorganization	Mean (*SD*)	2.28 (0.89)
PANSS excitation	Mean (*SD*)	1.91 (0.80)
PANSS depression	Mean (*SD*)	2.19 (0.92)

*PANSS, Positive and Negative Syndrome Scale; NOS, not otherwise specified. ^1^Two hundred and twelve participants reported years of education. ^2^One hundred and ninety-six participants reported age of onset and illness duration. ^3^Mean scores on the five PANSS factors were reported by 218 participants due to missing values (Lancon et al., 2000).*

### Confirmatory Factor Analysis

**FIGURE 1:**
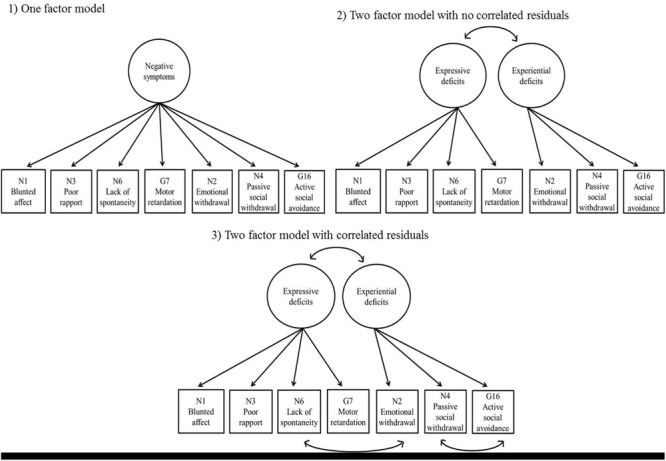
**Three models of the negative symptoms factor (NSF).** (1) One-factor model; (2) Two-factor model with no correlated residuals; (3) Two-factor model with correlated residuals. The circles at the top indicate latent factors; the boxes at the bottom indicate PANSS items connected to their corresponding factors. The arrows connecting boxes at the bottom indicate correlated residuals. For simplicity, the error terms of the PANSS items are not shown in this figure.

The one-factor model yielded the following fit index values: χ^2^ = 114.600, df = 14 (*p* < 0.001), NC = 8.186, CFI = 0.893, TLI = 0.839, RMSEA = 0.181 [CI: 0.151–0.212, 90%], and SRMR = 0.053, indicating poor fit. In contrast, the two-factor model exhibited substantially better data fit, except regarding its RMSEA value, which remained high: χ^2^ = 60.356, df = 13 (*p* < 0.001), NC = 4.643, CFI = 0.950, TLI = 0.919, RMSEA = 0.129 [CI: 0.097–0.162, 90%], and SRMR = 0.037. It should be noted that the two latent factors were closely correlated, *r* = 0.854. A third model was added with two correlated residuals between N2 (emotional withdrawal) and N6 (lack of spontaneity and flow of conversation) and between N4 (passive social withdrawal) and N16 (active social avoidance), as indicated by the modification indices (**Figure 1**). This third model exhibited the most satisfactory fit of the three: χ^2^ = 28.887, df = 11 (*p* < 0.01), NC = 2.626, CFI = 0.981, TLI = 0.964, RMSEA = 0.086 [CI: 0.048–0.125, 90%], and SRMR = 0.026. Chi-square difference tests comparing the three models found that the two-factor model with no correlated residuals exhibited significantly better fit than the one-factor model (χ^2^ = 54.244, df = 1, *p* < 0.001), and that the two-factor model with correlated residuals exhibited significantly better fit than the two-factor model with no correlated residuals, χ^2^ = 31.469, df = 2, *p* < 0.001. Standardized factor loadings of the observed variables (>0.40) indicated that latent factors in the third model were represented well by the observed variables; therefore, the third model was selected as the final model (**Table 2**).

**Table 2:** Standardized factor loadings in the third model.

	PANSS NSF-expressive	PANSS NSF-experiential
N1 Flat affect	0.85	–
N3 Poor rapport	0.81	–
N6 Lack of spontaneity and flow of conversation	0.83	–
G7 Motor retardation	0.58	–
N2 Emotional withdrawal	–	0.94
N4 Passive social withdrawal	–	0.82
G16 Active social avoidance	–	0.60

*PANSS NSF-expressive, the expressive factor of the negative symptom factor in the PANSS; PANSS NSF-experiential, the experiential factor of the negative symptom factor in the PANSS.*

### Correlation Analysis

The two NSF factors (expressive and experiential deficit) identified in the CFA exhibited non-significant correlations with age, gender, age of onset, illness duration, and years of education. Additionally, the experiential deficit factor of the NSF was significantly negatively correlated with total MAP-SR scores (*r* = -0.432, *p* < 0.001) and positively with BNSS expressive and experiential deficit scores (*r* = 0.560, *p* < 0.001; *r* = 0.706, *p* < 0.001, respectively; **Table 3**). The expressive deficit factor of the NSF was significantly negatively correlated with total MAP-SR scores (*r* = -0.248, *p* < 0.01) and positively with BNSS expressive and experiential deficit scores (*r* = 0.670, *p* < 0.001; *r* = 0.592, *p* < 0.001, respectively). We subsequently examined whether the two NSF factors exhibited specific relationships with subdomains of the BNSS and MAP-SR. The difference in correlation between the expressive deficits factor and the two factors (expressive and experiential deficits) of the BNSS was significant, *t*(217) = 2.19, *p* < 0.05. The difference in correlation between the experiential deficit factor of the NSF and the two factors of the BNSS was also significant, *t*(217) = -4.22, *p* < 0.001. Further, the difference in correlation between the two NSF factors and total MAP-SR scores was also significant, *t*(217) = 4.00, *p* < 0.001. Finally, the expressive deficits factor but not the social amotivation factor was correlated with poorer performance on the TMT-B (*r* = 0.214, *p* < 0.001).

**Table 3:** External validity with second-generation measures of negative symptoms.

	PANSS NSF-expressive composite score	PANSS NSF-experiential composite score
MAP-SR total	-0.248^∗∗^	-0.432^∗∗∗^
BNSS-expressive	0.670^∗∗∗^	0.560^∗∗∗^
BNSS-experiential	0.592^∗∗∗^	0.706^∗∗∗^
PANSS NSF-expressive composite	1	0.716^∗∗∗^
PANSS NSF-experiential composite	0.716^∗∗∗^	1
Cronbach’s alpha	0.847	0.860

*MAP-SR, Motivation and Pleasure Scale—-Self report; BNSS-expressive, the expressive factor of the Brief Negative Symptom Scale. MAP-SR (*n* = 141), BNSS (*n* = 78). ^∗∗^*p* < 0.01, ^∗∗∗^*p* < 0.001.*

### *Post hoc* Subgroup Analysis

To further explore the existence of clinical heterogeneity in groups with distinct profiles of negative symptoms dimensions, we identified two patient groups who had either relatively high (>3) expressive but low (≤3) experiential symptoms (*n* = 17, 7.727%) or low expressive but high experiential symptoms (*n* = 36, 16.364%) according to their NSF scores. We then compared the demographic and clinical characteristics of these two groups. The former group exhibited more severe mean overall negative symptoms than the latter group, *t*(50.833) = -2.389, *p* < 0.05. When controlling for overall negative symptom severity, the former group exhibited higher MAP-SR scores, *F*(1,30) = 13.638, *p* = 0.001, lower PANSS emotional symptoms, *F*(1,47) = 5.185, *p* < 0.05, and higher probability to smoke, *p* < 0.05.

## Discussion

This study examined the factor structure of the PANSS NSF in Korean individuals with chronic schizophrenia. It also provides novel information regarding the discriminant and convergent validity of sub-factors of the PANSS NSF with newly developed negative symptoms measurements (i.e., the BNSS and MAP-SR) and neurocognitive tasks.

The two-factor structure of the NSF exhibited significantly better data fit than the single-factor model, indicating that clinicians and researches may obtain more information on negative symptoms by using the two-factor structure. The two-factor model also exhibited adequate convergent and divergent validity with second-generation measures of negative symptoms. That is, although both NSF factors were correlated with the two subdomains of the BNSS (*r* > 0.05), the experiential NSF factor exhibited closer correlation with the experiential BNSS factor than with the expressive BNSS factor. Conversely, the expressive NSF factor was significantly more closely correlated with the expressive BNSS factor than with the experiential BNSS factor. Finally, MAP-SR scores were more closely correlated with the experiential NSF factor than with the expressive NSF factor.

The two-factor model’s fit was significantly improved when it was adopted with correlated residuals between N2 (emotional withdrawal) and N6 (lack of spontaneity and flow of conversation), and between N4 (passive social withdrawal) and G16 (active social avoidance). These correlated errors may reflect a common method effect, since item N2 and N6 share a rating method (i.e., behavioral observations during interviews). This would mean that low spontaneity and poverty of speech in the interviewee (N6) were sources of high scores on emotional withdrawal (N2). Item N4 and G16 also share a rating method (i.e., reports from families and staff) in addition to their shared content. This may lead to slight overestimation of the reliability of the experiential NSF factor, as clinicians and researchers are likely to use summed scores of items belonging to each factor, which do not account for correlated errors among items.

Regarding neurocognitive correlates of negative symptoms, we observed that longer completion time in TMT-B but not in the TMT-A was correlated with high expressive NSF factor scores. This finding may corroborate Cohen et al. (2013), who found that expressive deficits were related to performance on the Coding test but not to simple attentional ability as measured by the forward digit-span test in schizophrenia. This indicates that expressive deficits may be associated with impairments in cognitive functions such as cognitive flexibility and psychomotor abilities, which the TMT-B and Coding test both assess, possibly supporting the cognitive resource limitation model of diminished expressivity (Cohen et al., 2012). That model proposes that restricted expression in individuals with schizophrenia liability largely reflects depleted cognitive resources.

It should be noted, however, that the present study found close correlation between the two latent variables (i.e., the expressive and experiential factors) within the NSF. This suggests that the two dimensions measured by the NSF largely overlap with each other and may partly reflect a non-independent (though potentially dissociable) relationship between these dimensions, consistent with the current understanding of this symptom cluster (Blanchard and Cohen, 2006). Past studies have found that the two observed factors are correlated with a *r-*value of around 0.55 in the CAINS (Valiente-Gómez et al., 2015), 0.47 in the SANS (Mueser et al., 1994), and 0.65 in the PANSS (Fervaha et al., 2014). In addition to shared assumed variance between its two factors, the PANSS NSF may have a less differentiated factor structure due to its less extensive set of items, which cover a narrower range of negative symptom domains compared with instruments such as the SANS, CAINS, and BNSS (Daniel, 2013). Reliance on observable behavior during interviews when rating experiential deficits (e.g., N2, emotional withdrawal) may also contribute to this somewhat blurred structure, as indicated by the correlation of errors between N2 and N6.

Nonetheless, considering the results of the model comparison, the two-factor model seems to better represent the NSF than the conventional single-factor model, especially regarding sources of systematic error. It also seems to measure separable negative symptom dimensions to some degree, given the two NSF sub-factors’ different relationships with other negative symptom scales and cognitive tasks. This is in line with the growing consensus on the multi-dimensionality of negative symptoms. Researchers have long observed that negative symptoms have different aspects, e.g., the “weakening of the wellsprings of volition” and “restricted affect” (Bleuler, 1950; Kraepelin, 1971). It has also been noted that expressive deficits, or diminished facial, vocal, and bodily expressions, do not always accompany impoverished internal experience (Kring and Moran, 2008). Recent research has therefore examined the mechanisms of specific dimensions of negative symptoms such as blunted facial affect, poverty of speech, lack of anticipatory pleasure, and amotivation, rather than a single broad concept of negative symptoms (Strauss et al., 2013; Rocca et al., 2014; Hartmann et al., 2015; Kirschner et al., 2015).

The multi-factor solution has important clinical implications: it is able to acknowledge potential clinical heterogeneity among groups with differing profiles of specific symptom dimensions. For example, a significant portion of participants in the current sample was found to belong to either a high expressive/low experiential deficits (7.73%) or low expressive/high experiential deficits group (16.36%). These two groups were found to differ in psychiatric symptoms and proportion of smokers in our *post hoc* analysis. It has been reported that sub-dimensions of negative symptoms may have unique associations with functional outcomes, family history, illness course, and cognitive function. For instance, cross-sectional and longitudinal studies have found functional impairments to be more closely correlated with the experiential deficit domain than with the expressive deficit domain (Strauss et al., 2013; Ergül and Üçok, 2015), although Gur et al. (2006) reported close correlations with expressive deficits. Under the unitary concept of negative symptoms, valuable information on the etiology, mechanism, course, and treatment response of distinct negative symptoms may be lost (Rocca et al., 2014).

The present study has some limitations: It examined chronic patients with mild levels of psychiatric symptoms and who were taking antipsychotic medications. Therefore, this study’s results are not generalizable to patients who are unmedicated in different illness courses such as high clinical risk or first episode stage, or who have more severe symptomatology. Additionally, only two neurocognitive tasks were used in this study; future research should use comprehensive neurocognitive and social cognitive assessments to examine distinct cognitive correlates of the two negative symptom factors.

In sum, the current study indicates that the latent structure of the PANSS NSF is better represented by the two-factor model than by the one-factor model. Additionally, the two NSF factors exhibited adequate external validity in comparison with second-generation symptom measures. Measurement under the two-factor model of the NSF would be much improved if measurement errors possibly resulting from method variance were addressed. The current study provides a practical way to incorporate sub-dimensions of negative symptoms in clinical practice and research using the PANSS. Future research should further examine the sensitivity of this two-factor solution in predicting illness course, functioning, and treatment response associated with potentially discrete negative symptom dimensions.

## Author Contributions

SKJ and KHC designed the research. SKJ performed the literature search and statistical analyses, and wrote the first draft of the manuscript. HIC, EJJ, and GYL were responsible for assessment, data management, and supervision of the research assistants involved in this project at Korea University, and wrote the first draft of the methods section. YIC consulted on the statistical analyses. Subsequent drafts of the manuscript were edited by SKJ and KHC. All authors have contributed to and approved the final manuscript.

## Conflict of Interest Statement

The authors declare that the research was conducted in the absence of any commercial or financial relationships that could be construed as a potential conflict of interest.
